# How Good Are the Performances of Graphene and Boron Nitride Against the Wear of Copper?

**DOI:** 10.3390/ma14051148

**Published:** 2021-02-28

**Authors:** Min Cheol Kang, Hai Woong Park, Arnaud Caron

**Affiliations:** School of Energy Materials⋅Chemical Engineering, Korea University of Technology and Education, Cheonan 31253, Korea; reo031@koreatech.ac.kr (M.C.K.); hwpark@koreatech.ac.kr (H.W.P.)

**Keywords:** two-dimensional materials, metals, friction, wear, atomic force microscopy

## Abstract

We investigate the copper-wear-protective effects of graphene and boron nitride in single asperity sliding contact with a stiff diamond-coated atomic force microscopy (AFM)-tip. We find that both graphene and boron nitride retard the onset of wear of copper. The retardment of wear is larger with boron nitride than with graphene, which we explain based on their respective out-of-plane stiffnesses. The wear protective effect of boron nitride comes, however, at a price. The out-of-plane stiffness of two-dimensional materials also determines their friction coefficient in a wear-less friction regime. In this regime, a higher out-of-plane stiffness results in larger friction forces.

## 1. Introduction

Thin-film materials have long been used to enhance the tribological performances of substrate materials. For example, thin layers of soft metals, such as indium, have been used to lower friction forces due to their easy plastic deformation [1]. In contrast, hard coatings, such as diamond-like carbon (DLC) coatings, have been used to reduce abrasion, owing to their high hardness [2]. Depending on the coating’s relative hardness compared to the substrate, different governing mechanisms govern friction and wear (see References [3,4]). In soft coatings on harder substrates, two mechanisms can be identified: plowing and shearing. The thickness of the soft coating determines the prevalence of one over the other. As in ductile metals, the plowing mechanism consists of the penetration of a harder asperity into the softer counter material and the formation and motion of a pile-up ahead of the asperity. In the case of thin layers of a soft metal on a harder material, the penetration of asperity and the formation of a pile-up are limited by the film thickness. A transition from plowing to shearing is observed upon decreasing the thickness of the softer coating. In shearing, friction is determined by the strength of adhesive junctions (see also Reference [5]). The tribology of harder coatings on a softer substrate depends on the coating’s propensity to carry the load imposed by the counter body, which depends on both the coating thickness and its relative stiffness and hardness in comparison to the substrate [3,4].

With the development of miniaturized metal-based components and devices (see References [6,7]), new challenges regarding the reliability and damage prevention of metals at the nanometer scale need to be assessed. For microfabricated components operated in reciprocal motion, friction, stiction, and wear are significant challenges [8,9]. Conventional solutions, such as those introduced above, become inapplicable at the micro- and nanometer scales. While metals can be micro-manufactured, deposition of a conventional thin film, with a thickness of several tens to hundreds of nanometers, would compromise the geometry and the mechanical response of the whole micro-device.

In the last decades, extensive work has targeted the preparation of high-quality two-dimensional materials, such as graphene, hexagonal boron nitride (BN), or molybdenum disulfide (MoS_2_), by chemical vapor deposition (CVD) methods [10,11,12]. Notably, CVD has been used to grow single layers of graphene [10] and BN [11] on copper. The potential of graphene as a solid lubricant was soon recognized, and numerous studies have been published on the tribology of graphene (see References [13,14] and the references therein). Specifically, the lubricating effect of graphene is understood as arising from its chemical inertness and large in-plane stiffness [15]. In contrast to metals where the single nanoscale asperity sliding contact is governed by the formation of adhesive junctions and their shearing, the leading mechanism of wear-less friction of graphene has been identified as puckering [16,17,18]. The nanoscale wear protection of graphene grown on Pt (111) has been simulated and experimentally verified in Reference [19]. There, sudden jumps in the friction force signal were observed upon graphene rupture that was preceded by plasticity events in underlying platinum. The load-carrying effect of graphene grown on copper has further been simulated and measured during nanoindentation experiments [20,21]. In Reference [20], the authors performed molecular dynamic simulations of nanoindentation to better understand the effects of graphene on the plastic deformation of copper. It was found that graphene significantly strengthens the load-bearing capacity of copper, with the effect being proportional to the number of graphene layers. Furthermore, the authors explained their observations based on the impermeability of the graphene–copper interface to dislocations that inhibit the formation of slip steps on the surface. In Reference [21], the authors showed that the presence of graphene on copper reduces the average pop-in length during indentation and results in a more homogeneous distribution of dislocations below an indent and less prominent pile-ups around an indent. The authors further performed three-dimensional dislocation dynamics simulations. They showed that the experimentally observed effects could be rationalized based on the large back-stress induced by the graphene layer that acts as an impermeable barrier and leads to the delocalization of plasticity events. While these results agree with the ones presented in Reference [20], the authors in Reference [21] also found that the onset of plastic deformation of copper occurs at a lower load level in the presence of a graphene monolayer. This is in contrast to results in References [19,20].

Moreover, the intrinsic mechanical properties of suspended graphene and boron nitride have been investigated by AFM indentation and the pressurized blister test [22,23,24,25]. In Reference [22], the authors measured the elastic properties and the breaking strengths of free-standing unfolded monolayer graphene membranes by AFM indentation. The elastic response of graphene was interpreted within a nonlinear elasticity framework, yielding second- and third-order elastic stiffnesses of 340 N/m and −690 N/m, respectively; converting these values into three-dimensional elastic modules yields *E* = 1 TPa and *D* = −2 TPa. Moreover, the breaking strength was determined as 42 N/m (or *σ_f_* = 130 GPa). Similar experimental results have been reported in References [23,24] for defect-free BN. In Reference [24], the authors determined the two-dimensional Young’s modulus and breaking strength of a monolayer BN to be *E^2D^* = 290 N/m and *σ^2D^* = 23.6 N/m, respectively. From these results, defect-free graphene is thus stiffer and stronger than pristine BN. The effect of folding on the mechanical properties of free-standing graphene has been investigated by the pressurized blister test [25]. Owing to flexural phonons and static wrinkling, the authors in Reference [25] found that the effective stiffness of graphene at room temperature decreases to 20–100 N/m. A further key parameter to understand the mechanics of two-dimensional materials is the Föppl-von Kármán *γ*-number [26,27,28], which indicates the ratio between the in-plane stiffness and out-of-plane stiffness. In this framework, a large number corresponds to a membrane that can easily bend and crumple. Recently, the Föppl–von Kármán number of monolayer graphene was measured to be a thousand times higher than theoretically predicted; specifically, the author in Reference [28] found *γ* = 10^5^–10^7^. A corresponding quantification of *γ* has not yet been published for BN. However, a monolayer BN’s bending stiffness has recently been reported twice as high than that for monolayer graphene (see Reference [29]). In Reference [29], the authors further demonstrated how the difference in bending stiffness of both two-dimensional materials increases with the number of layers. This difference in the out-of-plane stiffness between graphene and BN may significantly affect the puckering mechanism of sliding friction.

Recently, nanoscale friction and wear of further two-dimensional materials transferred onto SiO_2_ substrates were investigated [30]. The authors showed that the tribological performances of SiO_2_ were substantially improved by all BN, MoS_2,_ and graphene. This observation was rationalized by their strong adhesion on the substrate. However, the damage characteristics of these atomically thin coatings were found to be distinct for each of them. As a general mechanism for atomically thin film failure, the authors identified the role of buckling of the thin film ahead of the sliding asperity, ultimately leading to the coating’s rupture. This work is representative of the wear-protective effect of two-dimensional materials on a hard substrate. A direct comparison of the effects of two-dimensional materials on softer substrates is, however, missing.

In this work, we investigate the wear performances of graphene and BN grown by CVD against the wear of copper by atomic force microscopy (AFM). The growth of graphene and BN on copper was confirmed by x-ray photoelectron spectroscopy (XPS). The friction and wear of copper and coated copper were measured by successively scanning a diamond-coated tip over the same area under the action of increasing normal forces. Therefore, both topographical changes and friction forces were recorded. For each of the three systems investigated here, two distinct regimes are observed in the load dependence of friction. The lower load regime corresponds to wear-less friction and is governed by the shearing of adhesive junctions for bare copper and puckering for graphene and BN. At larger normal force values, plasticity-mediated plowing of copper occurs. We find that graphene and BN retard the onset of plastic deformation of the copper substrate. This effect is larger for BN and is attributed to its larger out-of-plane stiffness.

## 2. Materials and Methods

A cold-rolled copper foil was purchased by Graphene Square, Republic of Korea, and a single layer of graphene on copper was prepared by chemical vapor deposition (see Reference [17] for preparation details). In addition, a monolayer hexagonal boron nitride-covered copper foil was purchased by GFM, Suwon-si, Korea. According to the manufacturers’ information, a single-layer BN was prepared on copper by chemical vapor deposition at 1300 K. For the sake of comparison, a cold-rolled copper foil sample was annealed in a mixture of Ar and H_2_ at 1300 K for 30 min.

**Figure 1:**
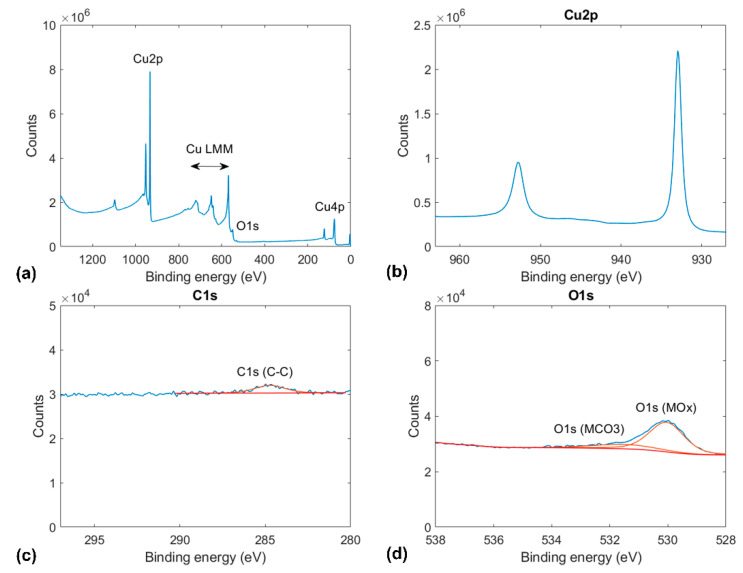
Indexed X-ray photoelectron spectroscopy (XPS) results for annealed copper: (**a**) full x-ray photoelectron spectrograms and spectrograms for the (**b**) Cu2p, (**c**) C1s, and (**d**) O1s orbitals.

**Figure 2:**
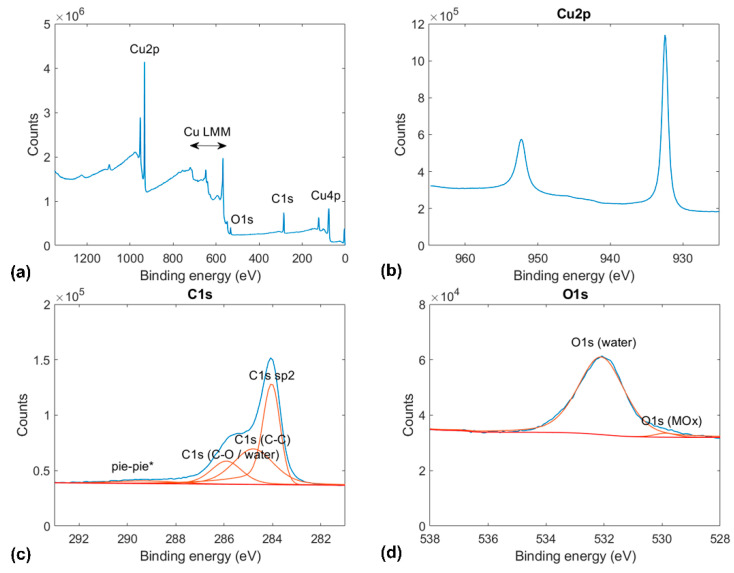
Indexed XPS results for graphene on copper: (**a**) full x-ray photoelectron spectrograms and spectrograms for the (**b**) Cu2p, (**c**) C1s, and (**d**) O1s orbitals.

**Figure 3:**
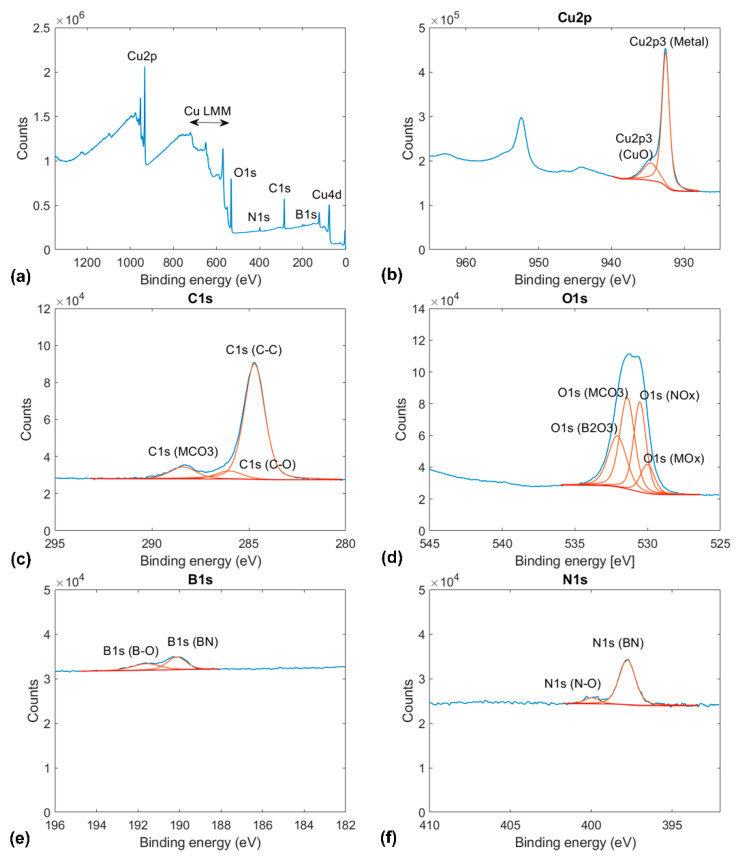
Indexed XPS results for boron nitride on copper: (**a**) full x-ray photoelectron spectrograms and spectrograms for the (**b**) Cu2p, (**c**) C1s, (**d**) O1s, (**e**) B1s, and (**f**) N1s orbitals.

Each sample was characterized by X-ray photoelectron spectroscopy (K-Alpha^+^ system, ThermoFischer Scientific, Waltham, MA, USA). The XPS measurements were recorded with a monochromated AlKα source (1486.6 eV, 12 kV) and a spot size of 400 mm. The measurements shown in Figure 1, Figure 2 and Figure 3 are the averages of 10-fold scan repetitions. Before the XPS measurements in ultrahigh vacuum (UHV) conditions (base pressure below 5 × 10^−9^ mbar), the samples were exposed to ambient conditions. To reduce the effect of surface contamination, a gentle Ar^+^ sputtering with low energy and current values was performed before XPS measurements on annealed copper (see Figure 1). In contrast, the XPS results for graphene and BN-coated copper shown in Figure 2 and Figure 3 were recorded without any preliminary sputtering to not damage the coatings.

Besides peaks corresponding to different copper orbitals, Figure 1 shows a clear O1s peak at 530 eV corresponding to copper oxide and a weaker peak at 532 eV corresponding to copper carbonate. In addition, XPS measurements of annealed copper revealed a weak C1s peak at 284.8 eV, corresponding to adventitious carbon contamination.

In addition to the same Cu peaks as above, the XPS results of graphene-coated copper, shown in Figure 2, exhibit a significantly higher C1s peak. This peak convolves a large sp2 contribution at 284.04 eV that corresponds to graphene and two smaller contributions of C–C bonds at 248.8 eV (adventitious carbon contamination) and C–O bonds at 286.5 eV, which has been reported for polycrystalline graphite wetted with water [31]. Furthermore, the XPS results for the graphene-coated copper sample exhibit a prominent O1s peak at 533 eV, corresponding to adsorbed water, and a weaker O1s peak at 530 eV than observed on annealed copper.

In the case of BN-coated copper, the same peaks corresponding to metallic copper, water, and adventitious carbon contamination as for annealed copper and graphene-coated copper are observed. The XPS results on BN-coated copper exhibit a contribution from copper oxide or copper carbonate to the peaks corresponding to the Cu2p and O1s orbitals. As expected, the XPS results on BN-coated copper exhibit clear peaks at 190.13 eV and 397.78 eV, corresponding to the contributions of BN bonds to the B1s and N1s orbitals. In the B1s and N1s orbital cases, weaker convoluted peaks at 191.6 eV and 399.96 eV can be observed. These peaks have previously been attributed to oxygen saturated defects in BN (see Reference [32]). Furthermore, the peak corresponding to the O1s orbital consists in the convolution of the following contributions: metal oxide (530.02 eV), adsorbed water (532.09 eV), B_x_O_y_ (191.6 eV), and NO_x_ (399.96 eV) [33,34].

Wear tests were performed in well-climatized and dehumidified laboratory conditions (*T* = 293 K and *RH* = 40%) by friction force microscopy (FFM) using an AFM XE-100 manufactured by Park Instruments, Republic of Korea. Therefore, a stiff diamond-coated AFM cantilever (CDT-NCLR, manufactured by NanoSensors, Switzerland) was used. The cantilever’s normal and lateral stiffnesses were calculated according to the geometrical beam theory by:

(1) $$C_{n}=\frac{Ewt^{3}}{4L^{3}}$$

(2) $$C_{l}=\frac{Gwt^{3}}{3h^{2}L}$$

where *E* is the Young’s modulus and *G* is the shear modulus. Therefore, the cantilever length *L* and width *w* were optically measured, and the tip height was set to *h* = 12 mm, according to the manufacturer’s data. The thickness of the cantilever *t* was determined according to:

(3) $$t=\frac{2\sqrt{12}\pi }{1.875^{2}}\sqrt{\frac{\rho }{E}}f_{0}L^{2}$$

where *f_0_* is the first free-bending resonance frequency of the cantilever and *ρ* is the mass density. Before the wear tests, the photodiode’s sensitivity *S* was calibrated by recording a force–distance curve on a noncompliant nanocrystalline diamond thin film and by extracting its slope 1/*S* in the range of repulsive forces. The wear tests consisted of repeated reciprocal sliding of the AFM tip with respect to the sample over a scan area *A_s_* = 2.5 × 2.5 μm^2^ with a velocity *v_s_* = 20 μm/s and under normal forces *F_n_* = 15 nN–6930 nN. Therefore, the scan areas were selected within single copper grains.

During the experiments, the topography and the lateral deflection signals were recorded in the forward and backward directions. The topographical images were used to calculate the evolution of the roughness parameter *R_q_* as a function of the normal force. The onset of wear for annealed copper and the degradation of graphene and BN were determined from the roughness evolution about the normal force. The lateral force *F_l_*-values were determined according to:

(4) $$F_{l}=\frac{3}{2}C_{l}\frac{h}{L}SV_{l}$$

where *V_l_* is the lateral voltage at the position-sensitive photodiode (PSPD). The friction force maps were calculated as:

(5) $$F_{f}=\frac{F_{l,fwd}-F_{l,bwd}}{2}$$

where *F_l,fwd_* and *F_l,bwd_* are the lateral force images recorded in the forward and backward directions, respectively. The mean friction value *<F_f_>* and its standard deviation σ*_<Ff>_* were calculated for each normal force value. After tribological tests, the topography of the scanned areas was recorded by amplitude-modulation noncontact AFM, and the average depth of the worn area was evaluated by plotting the height distribution of these images. All AFM results presented in this work were analyzed using a home-written MATLAB script [35].

## 3. Results

**Figure 4:**
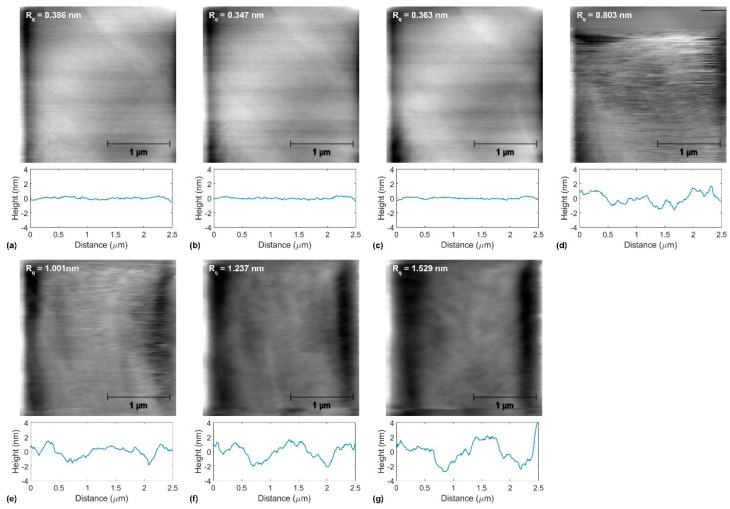
Topographical evolution during tribological tests of annealed copper at the normal force values (**a**) *F_n_* = 153 nN, (**b**) *F_n_* = 616 nN, (**c**) *F_n_* = 1232 nN, (**d**) *F_n_* = 2310 nN, (**e**) *F_n_* = 3850 nN, (**f**) *F_n_* = 5390 nN, and (**g**) *F_n_* = 6930 nN. In (**a**–**g**), the atomic force microscopy (AFM) topographical images at the normal force values indicated above, the corresponding roughness parameter *R_q_*-values, and representative line scans are presented.

**Figure 5:**
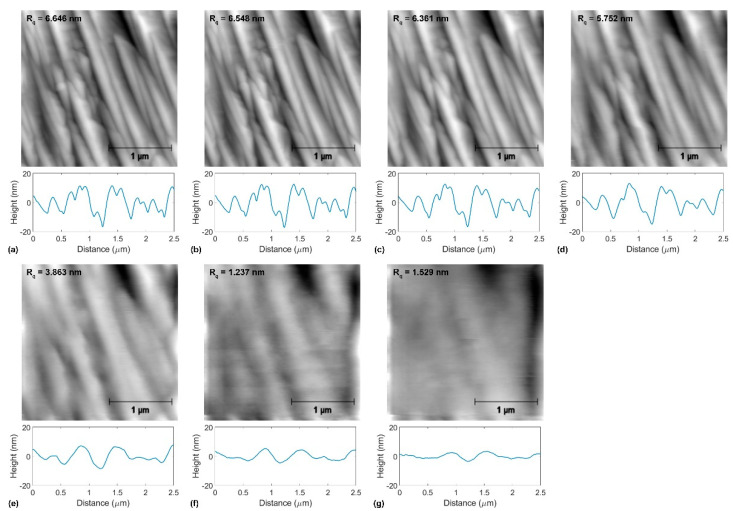
Topographical evolution during tribological tests of graphene-coated copper at the normal force values (**a**) *F_n_* = 153 nN, (**b**) *F_n_* = 616 nN, (**c**) *F_n_* = 1232 nN, (**d**) *F_n_* = 2310 nN, (**e**) *F_n_* = 3850 nN, (**f**) *F_n_* = 5390 nN, and (**g**) *F_n_* = 6930 nN. In (**a**–**g**), the AFM topographical images at the normal force values indicated above, the corresponding roughness parameter *R_q_*-values, and representative line scans are presented.

**Figure 6:**
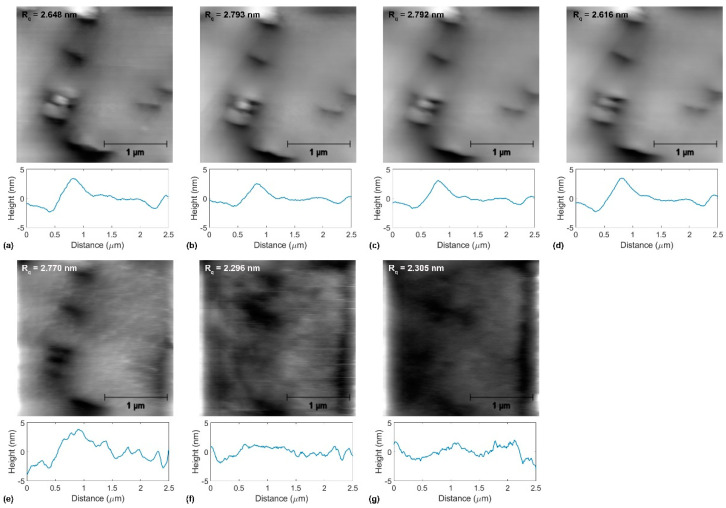
Topographical evolution during tribological tests of boron nitride (BN)-coated copper at the normal force values (**a**) *F_n_* = 153 nN, (**b**) *F_n_* = 616 nN, (**c**) *F_n_* = 1232 nN, (**d**) *F_n_* = 2310 nN, (**e**) *F_n_* = 3850 nN, (**f**) *F_n_* = 5390 nN, and (**g**) *F_n_* = 6930 nN. In (**a**–**g**), the AFM topographical images at the normal force values indicated above, the corresponding roughness parameter *R_q_*-values, and representative line scans are presented.

Figure 4, Figure 5 and Figure 6 show topographical images and representative topographical line scans recorded on annealed copper, graphene-coated copper, and BN-coated copper at the normal force values ranging from 153 nN to 6930 nN. In addition, the roughness parameter *R_q_* is indicated for each presented topographical image. The wear measurements consist of the recordings of 14 topographical and lateral force images in both the forward and backward directions with successively increased normal force values from 15 nN to 6930 nN. For the sake of clarity, Figure 4, Figure 5 and Figure 6 only show half of the recorded topographical images recorded in the forward direction. With increasing normal force values, the topographical images in Figure 4, Figure 5 and Figure 6 may appear fuzzier to the reader. This can be explained by the increased contact radius between the tip and sample surface while increasing the normal force that limits the lateral imaging resolution, on the one hand. On the other hand, with the onset of plastic deformation over a threshold normal force value, the surface topography irreversibly deviates from the original surface structure, as discussed for each sample below.

**Figure 7:**
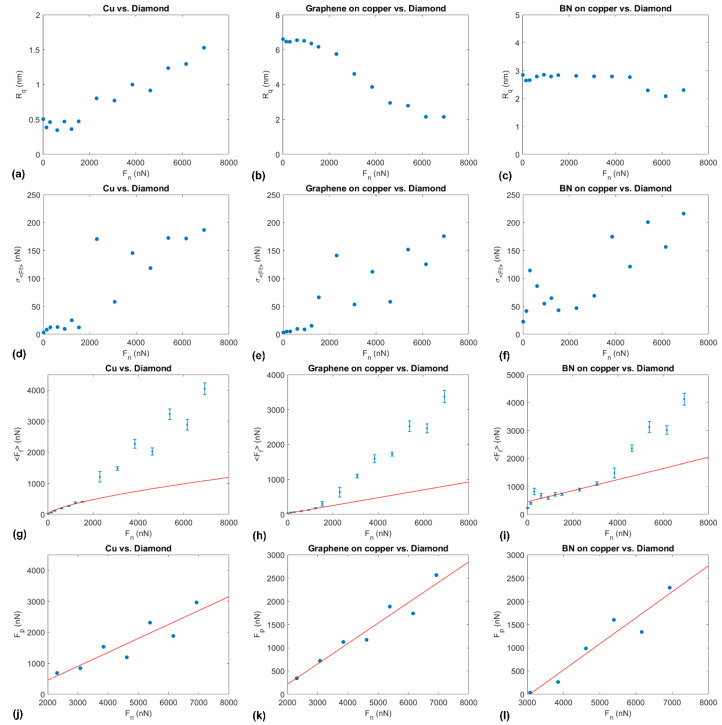
Evolution of (**a**–**c**) *R_q_*, (d–f) σ*_<Ff>_*, (**g**–**i**) *<F_f_>*, and (**j**–**l**) *F_p_* as a function of *F_n_* for (**a**,**d**,**g**,**j**) annealed copper, (**b**,**e**,**h**,**k**) graphene-coated copper, and (**c**,**f**,i,l) BN-coated copper. In (**g**), the solid red line illustrates the JKR fit function, while the solid red lines in (**h**–**l**) represent linear fit functions.

Figure 4 shows the evolution of topography for annealed copper upon tribological testing at the normal force values *F_n_* = 153 nN, 616 nN, 1232 nN, 2310 nN, 3850 nN, 5390 nN, and 6930 nN. We also indicate the roughness *R_q_* values for each topographical image in Figure 4. First signs of wear appear under a normal force value *F_n_* = 2310 nN (see Figure 2d). There, wear is accompanied by surface roughening, as indicated by the increase in the roughness parameter *R_q_* from ≈ 0.4 nm to ≈ 0.8 nm. Beyond this normal force value, the formation of ripples and a linear increase in the roughness parameter *R_q_* with *F_n_* were observed (see Figure 7a).

The graphene-coated copper surface investigated in this work was initially rougher than the bare copper surface (see Figure 5). We attribute this to the formation of folds upon cooling after graphenization. In the range of normal force values *F_n_* = 15 nN–923 nN, no noticeable topographical change can be observed, and the roughness parameter remains almost constant: *R_q_* = 6.6 nm–6.5 nm (see also Figure 7b). In the range of *F_n_* = 1232 nN–3850 nN, the graphene-coated copper’s topography is observed to be smooth. In this range of normal force values, the roughness parameter decreases down to *R_q_* = 3.863 nm. In this load regime, it appears that the graphene folds are gradually stretched. A further increase in the normal force values results in further smoothening of the surface until its initial features are lost; for *F_n_* = 6930 nN, we find *R_q_* = 2.151 nm. From these topographical measurements, no prominent rupture events of graphene can be identified.

For BN-coated copper, the surface topography appears mostly unchanged in the range of normal force values *F_n_* = 15 nN–3850 nN, within which *R_q_* = 2.8 nm. The first signs of wear appear as surface ripples at a normal force value *F_n_* = 4619 nN (see Figure 6e). Beyond this load, the coating appears to have disappeared, and the roughness parameter decreases from *R_q_* = 2.7 nm to 2.3 nm at *F_n_* = 6930 nN (see Figure 7c).

Figure 7 also shows a normal force dependence of the friction force mean value *<F_f_>* and standard deviation σ*_<Ff>_* for annealed copper, and graphene- and BN-coated copper. According to Bowden and Tabor, two mechanisms contribute to the friction of metals: shearing and plowing [5]. While plowing requires a threshold contact pressure to operate, shearing, i.e., the formation and deformation of adhesive junctions, operates from low contact pressure. Accordingly, the low normal force regime of friction of metals can be well fitted by the function:

(6) $$F_{s}\left(F_{n}\right)=\tau A_{c}\left(F_{n}\right)$$

where *F_s_* is the shearing force, *τ* is the shear strength between tip and sample surface,

(7) $$A_{c}=\pi a_{c}^{2}$$

is the contact area, and *a_c_* is the contact radius and depends on the normal force.

The contact area can be expressed by the Johnson–Kendall–Roberts (JKR) model [36], in which:

(8) $$a_{c}=\sqrt[{3}]{\frac{3}{4}\frac{R}{E^{*}}\left[\left(F_{n}+F_{ad}\right)+2F_{ad}+\sqrt{4F_{ad}\left(F_{n}+F_{ad}\right)+{\left(2F_{ad}\right)}^{2}}\right]}$$

where *R* is the tip radius, *F_ad_* is the adhesion force, and E^*^ is the reduced modulus of elasticity. For isotropic solids, E^*^ is given by:

(9) $$\frac{1}{E^{*}}=\left(\frac{1-\nu_{s}^{2}}{E_{s}}+\frac{1-\nu_{t}^{2}}{E_{t}}\right)$$

with *E_s,t_* and *ν_s,t_* being Young’s modulus and Poisson’s ratio of the sample and the tip, respectively [37]. Above a critical *F_n_*-value, the plowing mechanism becomes active and contributes to friction according to:

(10) $$F_{p}=\mu_{p}F_{n}$$

where *F_p_* is the plowing force and *m_p_* is the coefficient of plowing friction. Both shearing and plowing contributions add up to:

(11) $$F_{f}=F_{s}+{F_{p}|}_{F_{n}>F_{n_{y}}}$$

where $F_{n_{y}}$ is the onset value of the normal force for plowing.

The JKR model was used to fit the experimental results in the regime of low normal force values for annealed copper. In Figure 7g, the JKR model was fitted to the mean friction force values up to *F_n_* = 1539 nN using the MATLAB software package. The choice of this limit for the normal force values was motivated by the appearance of signs of abrasion in the topographical images for *F_n_* ≥ 2039 nN (see Figure 4d). Interestingly, the error bars’ width in Figure 7g, corresponding to the standard deviation from the mean friction force value shown in Figure 7d, becomes suddenly larger above *F_n_* ≥ 2039 nN. For the chosen interval of *F_n_*-values to fit the JKR model to our experimental friction values, the fit’s quality can be expressed in terms of its confidence *R^2^* = 0.9795. With *R* = 10 nm (in agreement with the manufacturer’s information for the tip roughness, i.e., the size of diamond nanocrystallites), *E_s_* = 130 GPa, *ν_s_* = 0.34, *E_t_* = 700 GPa, and *ν_t_* = 0.1, we obtain *τ* = 6.32 GPa and *F_ad_* = 0.2 nN. The obtained value *τ* = 6.32 GPa corresponds to $\tau =\frac{G}{7.59}$, where *G* = 48 GPa is the shear modulus of copper. The determined *τ*-value is thus close to the theoretical strength of copper. The plowing force *F_p_* was subsequently determined by extrapolating the fit function up to *F_n_* = 6930 nN and by subtracting the *F_s_*-values from *<F_f_>*. Figure 7j shows the normal force dependence of the plowing force. Fitting a linear function to these results, we find $F_{n_{y}}$ = 989 nN and *μ_p_* = 0.449. In this case, the confidence factor is *R^2^* = 0.8347.

In the case of graphene-coated copper, it is found that, in the regime of low normal force-values, the load-dependent friction is best fitted by a linear function (see Figure 7h). This is consistent with previous literature results, where the governing mechanism for wear-less friction of graphene-coated metal puckers [17,18]. As for annealed copper, the range of *F_n_*-values for fitting was selected for which no appreciable topographical change was observed (up to *F_n_* = 1232 nN; see Figure 5) and obtained the coefficient of puckering friction *μ* = 0.112 and *F_f0_* = 30 nN. The corresponding confidence factor is *R^2^* = 0.9833. Analogous to the case of annealed copper, the plowing friction force was calculated by extrapolating the fit function in the wear-less regime to larger *F_n_*-values and by subtracting it from the experimental friction force values (see Figure 7k). In this case, a linear dependence of the plowing friction force concerning the normal force is also observed and corresponds to *μ_p_* = 0.4386 and $F_{n_{y}}$ = 1507 nN. The confidence factor of fitting was determined to be *R^2^* = 0.9392. In the plowing regime, it is also noteworthy that the *F_f_* (*F_n_*) error bar-plot, i.e., the σ*_<Ff>_*-values, increases more rapidly than in the puckering regime, though the values are more scattered.

For BN-coated copper, two linear regimes of friction as a function of the normal force are also observed (see Figure 7i). In this case, however, the interval of normal force values to fit the wear-less friction regime was selected based on the σ*_<Ff>_*-values. This choice was motivated by the fact that the surface roughness parameter *R_q_* remained constant up to *F_n_* = 4500 nN while a clear change of slope in the *F_f_* (*F_n_*) plot is recognizable at a lower load. In Figure 7f, the σ*_<Ff>_*-values significantly increase for *F_n_* > 3079 nN. In the interval of *F_n_*-values between 15 nN and 3079 nN, we find a coefficient friction *μ* = 0.1989 and *F_f0_* = 453 nN. The confidence factor associated with this fit is far lower than for previous samples, at *R^2^* = 0.5059. As for the previous samples, the plowing force values were determined by extending the fit function for the low load regime to higher *F_n_*-values and by subtracting it from the experimental friction force values. Here also, *F_p_* is found to increase linearly with the *F_n_*. Fitting this dependence with a linear function, we find *μ _p_* = 0.5612 and $F_{n_{y}}$ = 3066 nN (see Figure 7l). For this fit, the confidence factor is *R^2^* = 0.9144.

**Figure 8:**
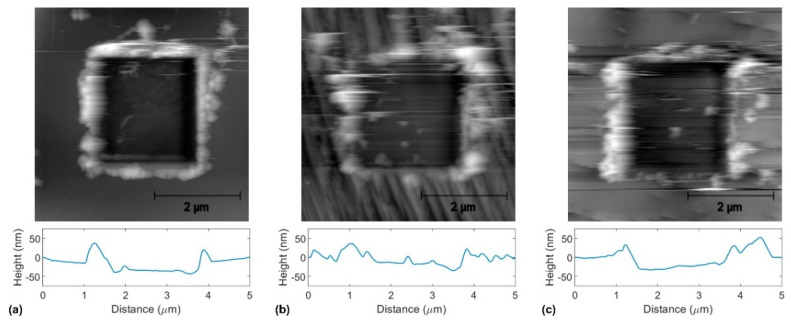
Topographical images recorded by noncontact AFM after tribological testing of (**a**) annealed copper, (**b**) graphene-coated copper, and (**c**) BN-coated copper.

Figure 8 shows topographical images recorded by noncontact AFM after tribological tests. We observe the worn area as a square with depreciated grey-scale values corresponding to lower height values in all three cases. All three images in Figure 6 also exhibit major pile-ups around the scanned area, thus confirming plowing as the governing wear mechanism.

**Figure 9:**
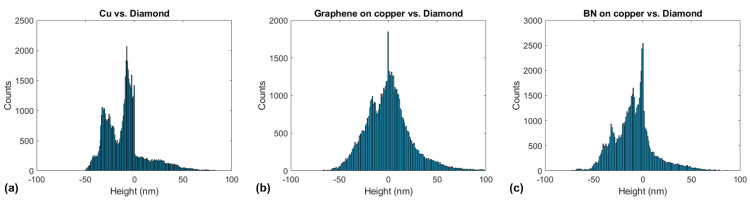
One-dimensional height distribution corresponding to the topographical images in Figure 6 for (**a**) annealed copper, (**b**) graphene-coated copper, and (**c**) BN-coated copper.

Figure 9 shows the one-dimensional height distributions corresponding to the topographical images in Figure 8. We observe a prominent peak centered around 0 nm and a second peak corresponding to each case’s scratched area. In the case of annealed copper, the mean wear-depth is *δ_w_* = 30 nm, while for graphene-coated and BN-coated copper, they are *δ_w_* = 16 nm and *δ_w_* = 9 nm, respectively.

## 4. Discussion

The tribological response observed for annealed copper in this work agrees with observations made on annealed copper in Reference [17] or metallic alloys in Reference [37]. In Reference [17], we observed that annealed copper’s shear strength varied between 7 GPa and 2 GPa depending on the sliding velocity. In Reference [38], we found the shear strength of an Ag–Cu nano-eutectic alloy to be *τ* = 12 GPa. Similarly, we find for copper *τ* = 6.3 GPa in this work. Furthermore, we observe that plowing becomes active beyond the normal force value $F_{n_{y}}$= 989 nN. Using the expression of the contact radius according to the JKR model, one obtains ${a_{c}|}_{F_{n_{y}}}$= 3.93 nm. Correspondingly, the contact pressure at the onset of plowing can be calculated as:

(12) $$p_{y}=\frac{F_{n_{y}}}{\pi {a_{c}|}_{F_{n_{y}}}{}^{2}}\;=\;3.5\;\mathrm{GPa}$$

Since the underlying mechanism for plowing is plastic deformation, it is interesting to compare the *p_y_*-value with copper’s hardness. According to the model developed by Nix and Gao to account for the depth dependence of metals’ hardness,

(13) $$\frac{H}{H_{0}}=\sqrt{1+\frac{\delta^{*}}{\delta }}$$

where *H_0_* is the hardness value in the absence of geometrically necessary dislocations, and d^*^ characterizes the depth dependence of the hardness and depends both on the indenter geometry and *H_0_* [39]. In Reference [39], the authors reported that *H_0_* is ≈0.3 GPa and δ^*^ is ≈ 0.5 mm for copper. In this work, the indenter’s penetration depth at the onset of plowing can be estimated according to:

(14) $${\delta |}_{F_{n_{y}}}=\frac{{a_{c}|}_{F_{n_{y}}}{}^{2}}{R}\approx 1.54\mathrm{nm}$$

This yields the hardness value *H* = 4.7 GPa, which is relatively close to the calculated value of the contact pressure at the plowing onset.

Comparing these results for annealed copper with graphene- and BN-coated copper, it is found that the shearing friction model does not hold in both cases. This observation confirms recent results on the wear-less friction of graphene and can be explained by the chemical inertness of both graphene and BN, which prevents the formation of adhesive junctions with the indenter [17,18,30]. Instead, the governing mechanism for wear-less friction of graphene has been identified to be puckering. A graphene fold forms at the leading edge of the indenter and moves ahead of the indenter while it is being sledded. Therefore, the friction force associated with puckering of graphene is a result of its high in-plane stiffness [18] and its low out-of-plane stiffness [28,29]. It is understood that the formation and motion of a fold ahead of the indenter is made easier for a low out-of-plane stiffness or a high ratio between the in-plane stiffness and the out-of-plane stiffness, as expressed by the Föppl–von Kármán number. For this sample, the onset of plowing occurs at $F_{n_{y}}$ = 1507 nN. This value is significantly larger than for annealed copper. It is noteworthy that, for graphene-coated copper, no rupture event of graphene could be observed. Instead, above *F_n_* = 1500 nN, it is observed that graphene folds gradually smooth out, indicating that an AFM diamond tip stamps graphene into copper. For graphene-coated copper, the plowing friction coefficient value is almost identical to the value found for annealed copper. This evidences that, in this case too, the plowing forces arise from plastic deformation of the underlying copper substrate. Graphene does not suppress plastic deformation of the copper substrate but retards it. As a result, the average wear depth is observed to decrease from 30 nm for annealed copper down to 16 nm for graphene-coated copper. These results agree with the observations reported in Reference [20]. There, the authors simulated the indentation response of graphene-coated copper and found that, in load-controlled conditions, the penetration depth of the indenter decreased for graphene-coated copper compared to bare copper.

In BN-coated copper, the wear-less friction is characterized by a slightly larger friction coefficient *μ* = 0.19 than on graphene, where *μ* = 0.11. In this case, too, the governing mechanism of wear-less friction is assumed to pucker. The larger corresponding friction coefficient can be explained by a larger out-of-plane stiffness of BN compared to graphene. In Reference [29], the out-of-plane stiffness of BN was reported to be twice as high as for graphene. For this system, the wear-less regime extends to a normal force value $F_{n_{y}}$ = 3066 nN, beyond which plastic deformation-mediated plowing of the underlying copper substrate becomes active. The corresponding coefficient of plowing friction is *μ_p_* = 0.56 and is slightly larger than that for annealed copper and graphene-coated copper. In the range of normal force values *F_n_* = 3079 nN–3850 nN, no noticeable topographical changes are observed. As for graphene, it is assumed that the BN layer is stamped into copper. At *F_n_* = 4619 nN, fine ripples on the topography of BN-coated copper become visible. These ripples seem to be precursors of the BN layer rupture since we observe significant topographical changes beyond this normal force value. Although BN appears to rupture above *F_n_* = 4619 nN, its wear-protective effect is larger than that for graphene-coated copper and is characterized by a lower average wear depth *δ_w_* = 9 nm. The larger load-bearing capacity of BN compared to graphene can be explained by the larger out-of-plane stiffness of BN that leads to its higher resistance to be stamped into copper.

Thus, both graphene and BN retard the onset of plastic deformation-mediated plowing of copper. Both graphene and BN layers act as load supports. The more pronounced effect of BN is attributed to its larger out-of-plane stiffness than graphene. While a larger out-of-plane stiffness enhances the wear-protective effect of two-dimensional materials, it is detrimental to their performance as a solid lubricant.

## 5. Conclusions

We investigated copper’s wear-protective effects by graphene and boron nitride in single asperity sliding contact with a stiff diamond-coated AFM tip. In the wear-less regime, shearing dominates the friction of bare copper. In contrast, the wear-less friction of graphene- and BN-coated copper is governed by puckering. Both graphene and boron nitride were found to retard the onset of wear of copper. This effect is larger for BN than graphene. The retarding effect on wear is attributed to the load-bearing capacity of graphene and BN, although the coefficient of plowing friction is not affected by the coatings. This indicates that the wear mechanism consists of plastic deformation of copper in all three investigated systems. Owing to its larger out-of-plane stiffness, BN exhibits a more pronounced wear protective effect than graphene. For the same reason, though, BN is a less effective solid lubricant than graphene.

## Data Availability

The data presented in this study are available on request from the corresponding author.
